# A rare case of advanced pelvic organ prolapse concurrent with high-grade rectal prolapse: A case report

**DOI:** 10.1097/MD.0000000000043648

**Published:** 2025-08-01

**Authors:** Marian Botoncea, Călin Molnar, Cosmin-Lucian Nicolescu, Cătălin Baltă, Vlad-Olimpiu Butiurca, Orsolya Martha, Claudiu Varlam Molnar

**Affiliations:** a Surgical Clinic No. 1, Emergency County Hospital Târgu Mureș, “G.E. Palade” University of Medicine, Pharmacy, Science and Technology of Târgu Mureș, Târgu Mureș, Romania; b Surgical Clinic No. 1, Emergency County Hospital Târgu Mureș, Târgu Mureș, Romania; c Urology Clinic, County Clinical Hospital Târgu Mureș, “G.E. Palade” University of Medicine, Pharmacy, Science and Technology of Târgu Mureș, Târgu Mureș, Romania; d Obstetrics and Gynecology Clinic, Emergency County Hospital Târgu Mureș, “G.E. Palade” University of Medicine, Pharmacy, Science and Technology of Târgu Mureș, Târgu Mureș, Romania.

**Keywords:** combined treatment, pelvic organ prolapse, rectal prolapse, rectopexy, sacrocolpopexy

## Abstract

**Rationale:**

Concurrent pelvic organ prolapse (POP) and rectal prolapse is a rare condition that poses significant therapeutic challenges. A customized, multidisciplinary strategy is frequently required, particularly in complex cases with underlying functional deficits.

**Patient concerns:**

A 71-year-old woman presented with stage III POP and grade V rectal prolapse. Her history included no pregnancies or vaginal intercourse, and she previously underwent sacral tumor excision resulting in anal and urinary incontinence, requiring long-term catheterization.

**Diagnoses:**

Preoperative clinical examination and imaging (transabdominal and transvaginal ultrasound) confirmed the diagnosis of stage III POP and grade V rectal prolapse. Routine blood tests, vaginal bacteriological cultures, and Pap smear were unremarkable.

**Interventions:**

The patient underwent a multidisciplinary surgical procedure including hysterectomy, sacrocolpopexy, and rectopexy using a Pro-Grip™ self-fixating mesh. The mesh was placed to anchor the vaginal stump and provide rectal support simultaneously.

**Outcomes:**

The patient had an uneventful recovery, with drain removal on postoperative day 5 and discharge on day 7. Follow-up at 3 months, 6 months, and 1 year showed no recurrence of prolapse. The patient declined additional imaging at 1 year, considering herself fully recovered.

**Lessons:**

This case demonstrates that a self-fixating mesh can provide effective concurrent repair of POP and rectal prolapse in a single procedure. Short-term outcomes were favorable, but long-term follow-up remains essential to assess durability and potential complications.

## 1. Introduction

Pelvic organ prolapse (POP) and rectal prolapse are distinct but occasionally coexisting conditions that can significantly impact quality of life.^[1,2]^ POP is characterized by the descent of pelvic organs into the vaginal canal,^[3]^ whereas rectal prolapse involves the protrusion of either the mucosal layer or the full-thickness rectal wall through the anus.^[2]^ Risk factors for POP include advanced age, multiple pregnancies, obesity, and prior hysterectomy.^[3,4]^ Simultaneous presentation of both conditions is rare, and no “gold standard” treatment has been defined.^[5]^ This case report details a patient presenting with POP with cysto-rectocele, as well as rectal prolapse. The condition was successfully repaired through an abdominal surgical approach using a self-fixating mesh.

## 2. Case report

A 71-year-old normosthenic female presented with a 2-year history of progressive pelvic discomfort, persistent lower abdominal pressure, and visible prolapse. The patient reported no history of vaginal intercourse or pregnancies. Her medical history included anal and urinary incontinence following the surgical removal of a bony tumor from the sacrum adherent to the rectum at age 16. Histopathological examination at that time identified the tumor as a giant cell tumor of the bone. Postoperatively, she required permanent urinary catheterization due to long-standing urinary incontinence and voiding dysfunction. Additionally, she had a history of right acoustic neuroma, for which she underwent a right retromastoid craniotomy in March 2014, resulting in complete tumor excision. However, the facial and vestibulocochlear nerves (VIIth and VIIIth cranial nerves) could not be preserved, leading to right-sided facial paralysis and deafness. The patient reported no history of smoking or significant alcohol consumption.

**Figure 1.**
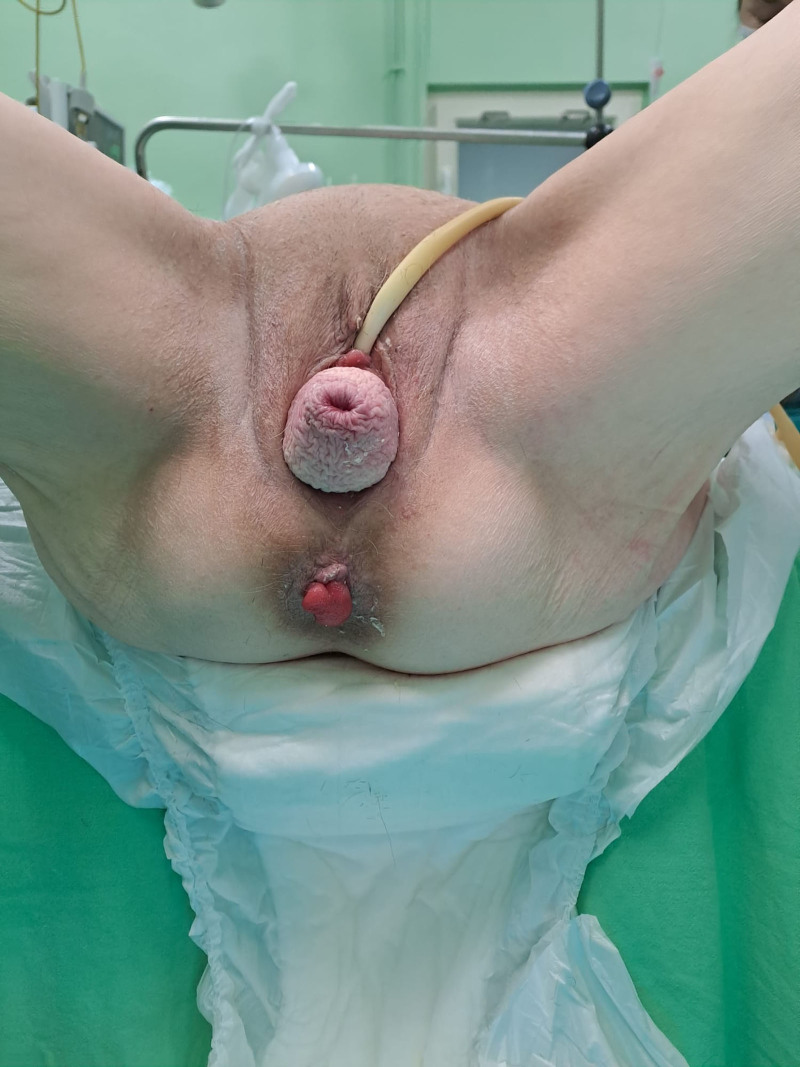
Concomitant stage III POP and grade V rectal prolapse. POP = pelvic organ prolapse.

Preoperative clinical examination, along with transabdominal and transvaginal evaluation performed on November 22, 2023, in a private clinic and repeated on January 16, 2024, in the Gynecology Department, confirmed the diagnosis of stage III POP with cysto-rectocele, as classified by the POP quantification staging system,^[6]^ and a grade V rectal prolapse, based on the Oxford rectal prolapse grading system (Fig. 1),^[7]^ with no signs of mucosal ulceration. Although ultrasound examinations were performed, the corresponding images are unavailable for publication. Only the written reports were accessible and used in this case. Routine preoperative blood tests, bacteriological cultures from vaginal secretions, and Pap smear revealed no evidence of infection or abnormal cytology.

**Figure 2.**
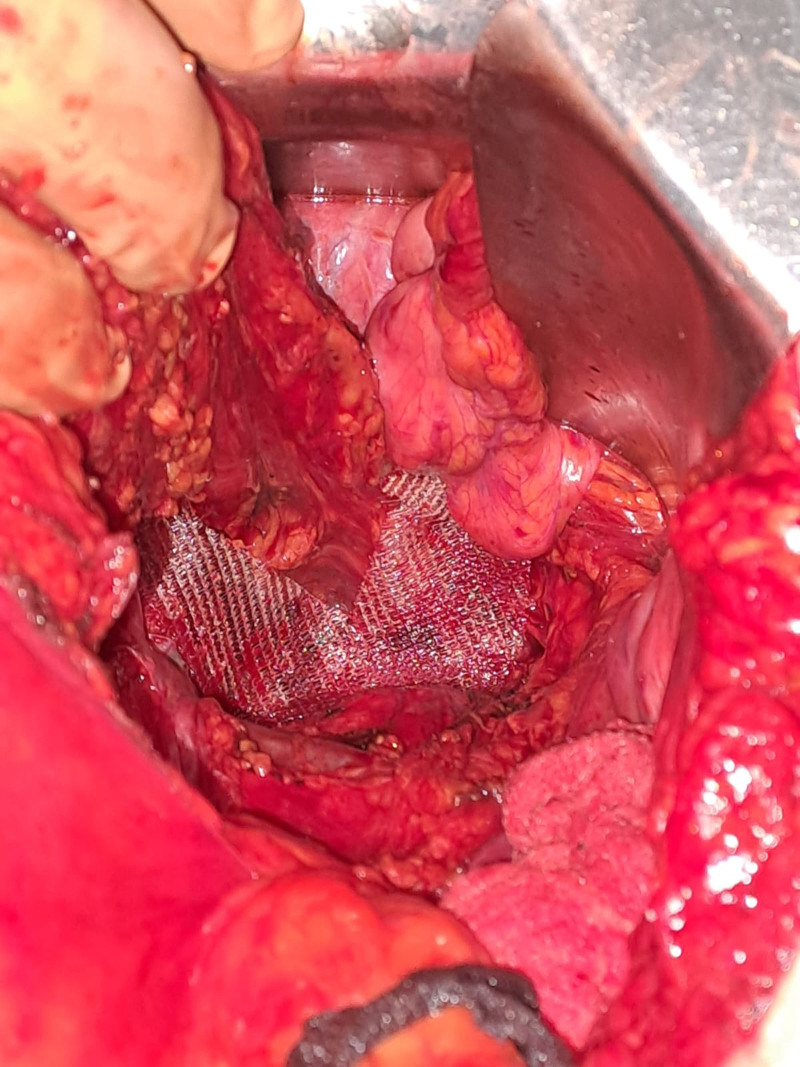
Mesh embracing the rectum and mesorectum, attached to the vaginal stump and sacrum.

**Figure 3.**
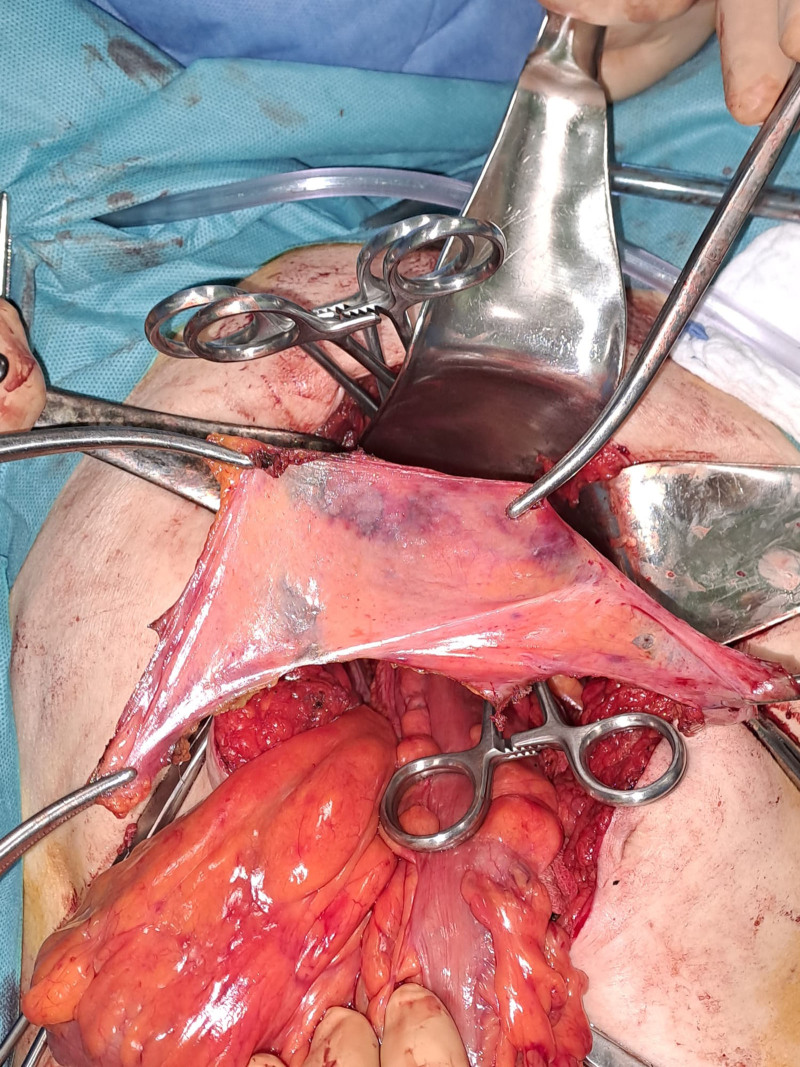
Peritoneum of the Douglas pouch.

**Figure 4.**
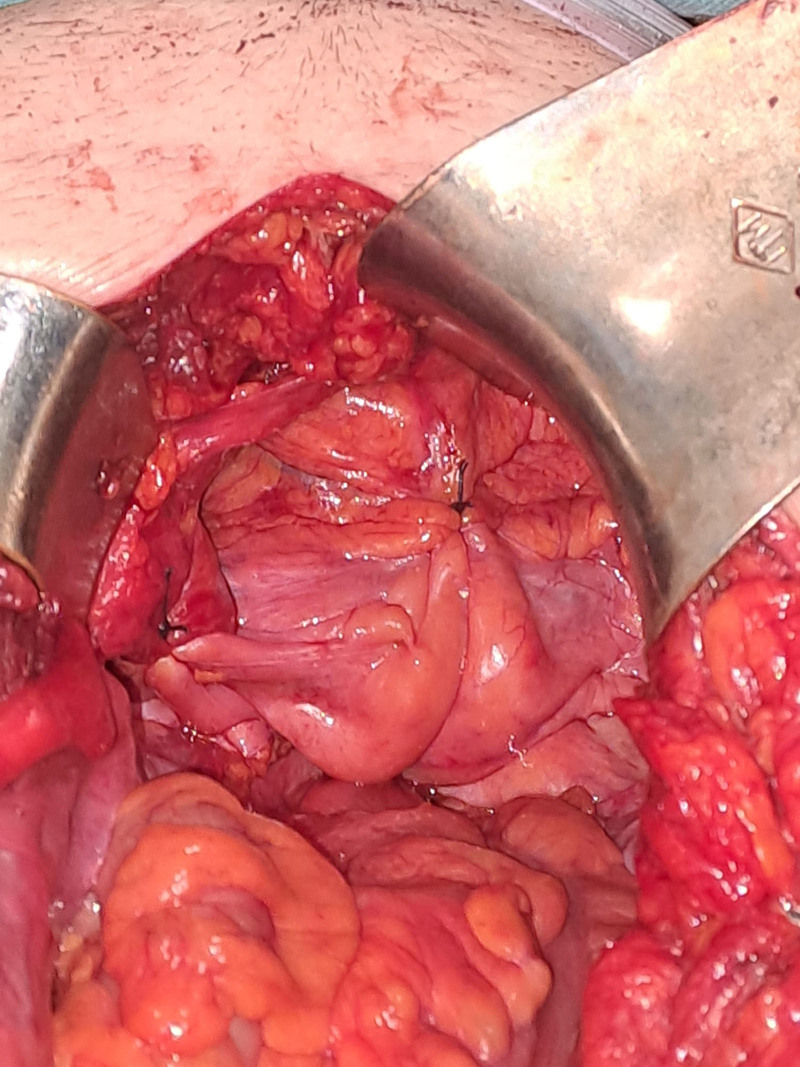
Peritonization of the mesh.

Following adequate preoperative preparation, which included manual reduction of the rectal prolapse, administration of laxatives 24 hours before surgery, and prophylactic administration of cefazolin 30 minutes before the operation, surgical intervention was performed on January 18, 2024 (operative record: 2049/2024) by a multidisciplinary team. The procedure involved an exploratory laparotomy, total extracapsular hysterectomy using the Wiart technique with bilateral adnexectomy, resection of the upper third of the vagina, sacrocolpopexy, and rectopexy using a Pro-Grip™ self-fixating mesh (Fig. 2).^[8]^ Additional procedures included excision of the peritoneum from the Douglas pouch (Fig. 3), anterior cystopexy, dual drainage of the sacral cavity, peritonization of the self-fixating mesh (Fig. 4), single-layer laparorraphy, and skin suturing.

The dual-layer mesh features a central orifice that is enlarged to accommodate the diameter of the rectum and mesorectum. The 2 arms of the mesh encircle the rectum and mesorectum, anchoring onto the vaginal stump, while the fixed portion adheres to the promontory or presacral fascia. This configuration enables colpopexy, with the rectum positioned on the mesh as though resting in a hammock.^[8]^

**Figure 5.**
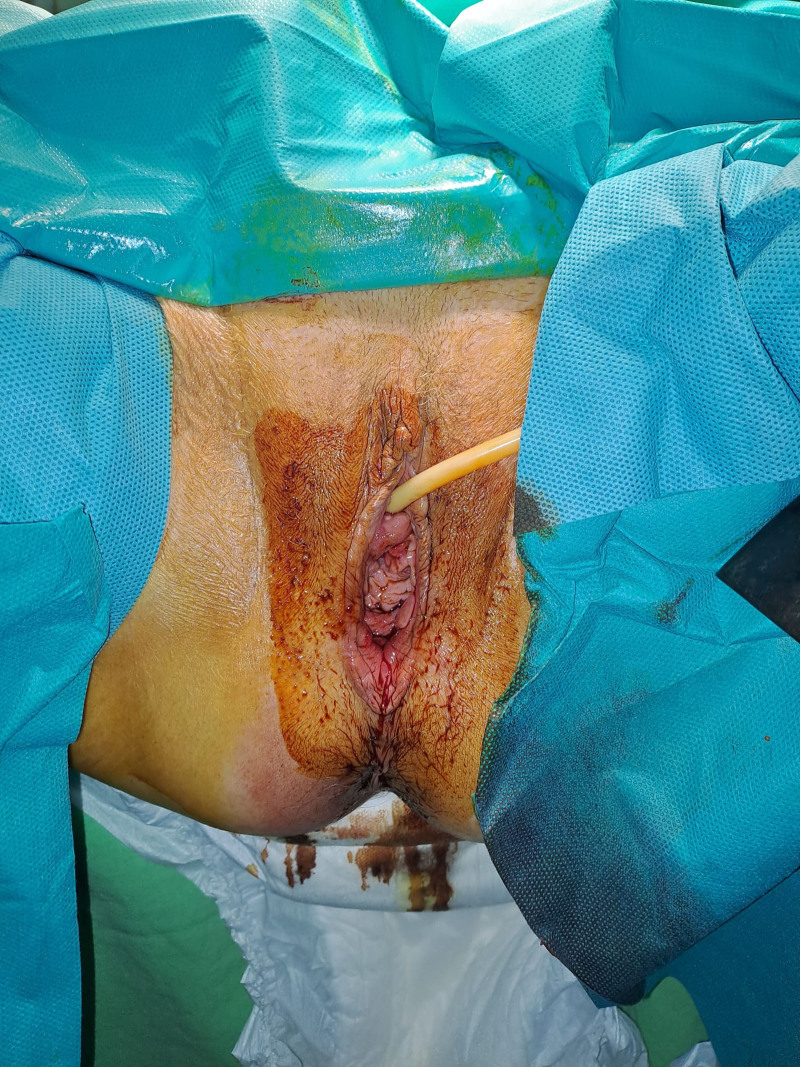
Immediate postoperative result.

The surgical procedure was performed without any intraoperative incidents or complications. The immediate postoperative outcome is shown in Figure 5.

Postoperative management included daily wound care and early mobilization. Prophylactic antibiotic therapy was continued for 24 hours following the procedure. Abdominal drains were monitored daily and removed without complications on postoperative day 5. Pain control was achieved through a multimodal analgesic regimen, and oral intake was resumed once bowel function was restored. The patient was discharged on postoperative day 7 with recommendations to avoid heavy lifting, strenuous activity, and prolonged standing for at least 6 weeks.

**Figure 6.**
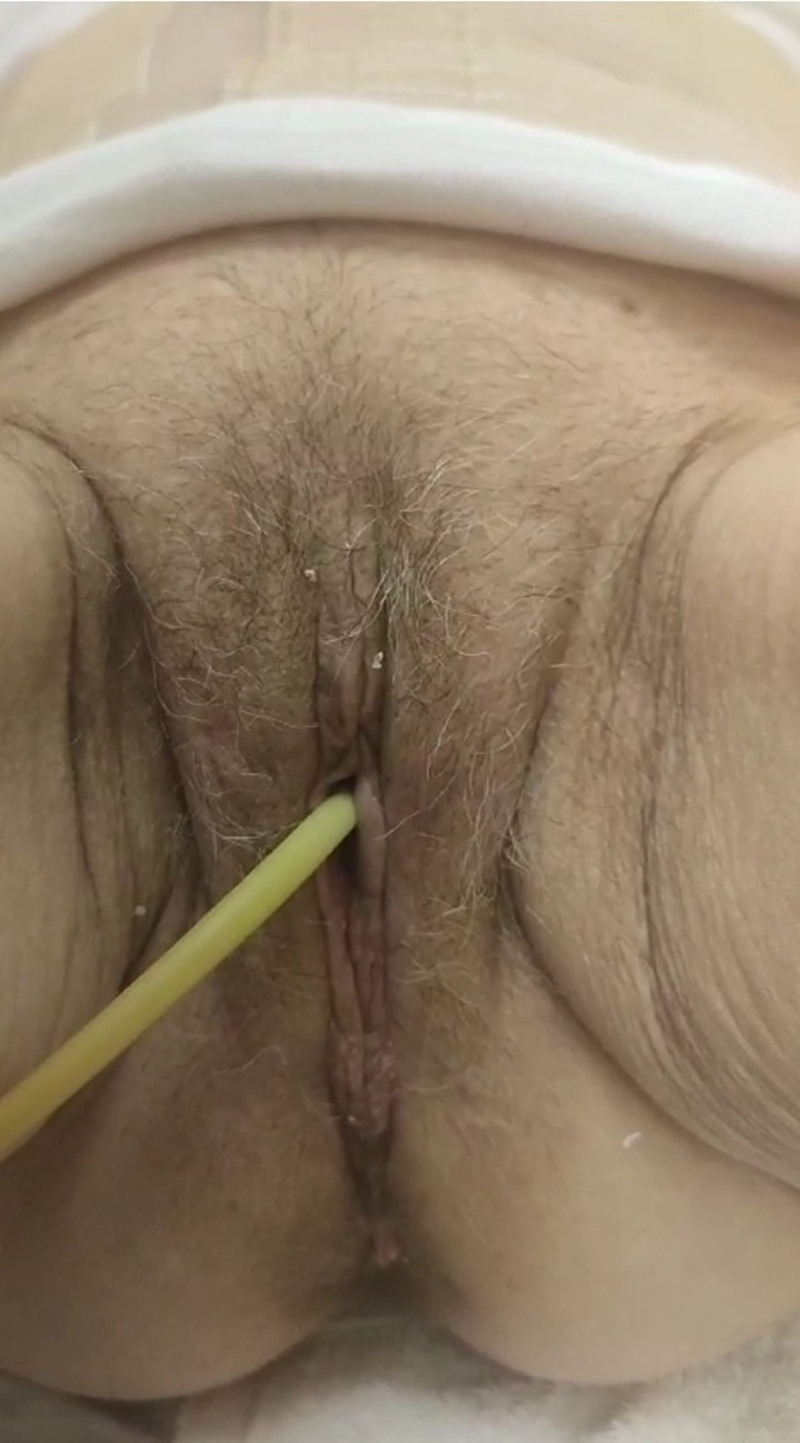
Postoperative result at 3 mo.

**Figure 7.**
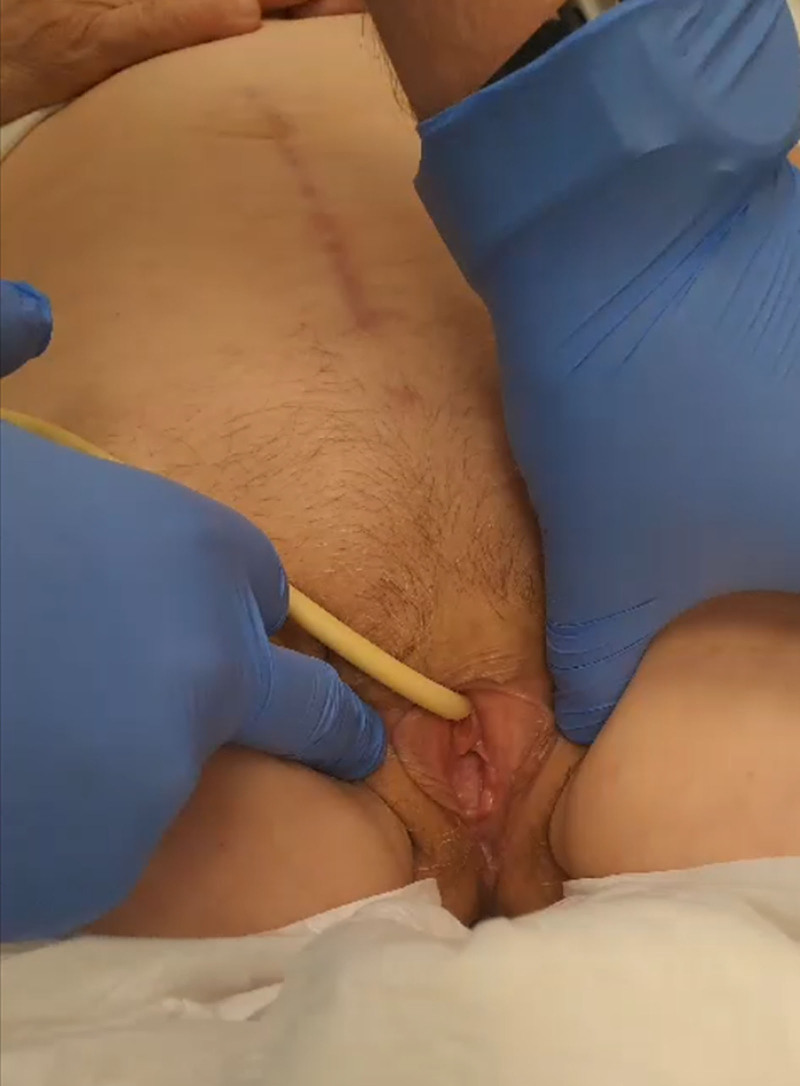
Postoperative result at 6 mo.

The postoperative course was uneventful, with no recurrence of prolapse observed at follow-up assessments 3 months (Fig. 6), 6 months (Fig. 7), and 1 year after surgery.

Ethical approval was not required for this case report because it describes a single patient and does not involve research procedures. The patient gave written informed consent for the publication of clinical details and images.

## 3. Discussion

POP and rectal prolapse are common among older adults and can significantly impact quality of life.^[3,7]^ Although these conditions often occur independently, studies have shown that up to 31% of women with rectal prolapse also present with POP, including vaginal prolapse and urinary incontinence.^[9]^ This case report describes a rare presentation and demonstrates an alternative abdominal surgical approach using a self-fixating mesh to address both conditions simultaneously.

The patient’s medical history, particularly the prior sacral tumor excision leading to long-standing urinary incontinence, likely contributed to the development of these conditions. Notably, the absence of common risk factors such as multiparity or vaginal delivery underscores the complexity of her presentation, suggesting that prior anatomical disruption from surgery can predispose patients to prolapse.

Due to the uncommon coexistence of POP and rectal prolapse, no established “gold-standard” surgical approach exists for their management.^[5,9]^ This case highlights the importance of an individualized, multidisciplinary approach. The dual-layer self-fixating mesh enabled the combination of colpopexy and rectopexy by securely anchoring to the sacrum and vaginal stump while supporting the rectum in a “hammock” configuration. This technique limited mesorectal mobilization while eliminating the need for anastomosis and reducing the risks associated with auxiliary fixation methods.

The patient’s favorable postoperative outcome, with no evidence of recurrence at 3, 6 months (Figs. 6, 7), and 1 year, highlights the efficacy of this approach. At 1-year follow-up, the patient declined further photographic documentation, stating she considered herself fully recovered and no longer wished to undergo additional clinical photography. However, long-term follow-up remains essential to monitor for potential complications such as mesh erosion, infection, or late-onset prolapse recurrence.

Laparoscopic or robotic procedures for the simultaneous management of rectal prolapse and POP have been increasingly reported with favorable outcomes.^[4]^ In 2020, Campagna et al^[10]^ published a prospective cohort study involving 98 patients who underwent ventral rectopexy and laparoscopic sacrocolpopexy for concurrent prolapse. The study reported no intraoperative complications, no conversions to open surgery, no recurrence of rectal prolapse, and only 1 recurrence of POP, which did not require surgical intervention.^[10]^

Similarly, a retrospective review by Bordeianou et al^[11]^ suggested that middle compartment suspension performed during the same procedure as rectal prolapse repair may improve the short-term durability of the repair. Wallace et al,^[12]^ in a retrospective cohort study, found that combined POP and rectal prolapse surgery resulted in postoperative complication rates like those observed in patients undergoing POP repair alone within 30 days of surgery. Additionally, the recurrence rate of POP was comparable between the combined procedure and isolated POP repair.^[12]^

The multidisciplinary management approach detailed in this case report highlights the utility of an auto-fixating mesh for the combined surgical treatment of POP and rectal prolapse. Long-term follow-up remains essential to assess the durability of this technique and to monitor for potential complications.

## 4. Conclusion

This case highlights the successful multidisciplinary surgical management of a rare combination of stage III POP and grade V rectal prolapse in a patient with a unique history of sacral tumor resection and pelvic floor dysfunction. The use of a self-fixating mesh in a single-stage procedure combining hysterectomy, sacrocolpopexy, and rectopexy provided good results, with no recurrence at 1-year follow-up. Continued follow-up remains essential to evaluate long-term outcomes and potential mesh-related complications.

## Acknowledgments

The authors thank the patient for providing informed consent for publication of this case report and associated images.

## Author contributions

**Methodology:** Marian Botoncea.

**Supervision:** Calin Molnar.

**Writing – original draft:** Cosmin-Lucian Nicolescu, Cătălin Baltă, Vlad-Olimpiu Butiurca.

**Writing – review & editing:** Orsolya Martha, Claudiu Varlam Molnar.
